# Intestinal Microbiota and Gene Expression Alterations in Chinese Mitten Crab (*Eriocheir sinensis*) Under Deltamethrin Exposure

**DOI:** 10.3390/antiox14050510

**Published:** 2025-04-24

**Authors:** Chunyi Zhong, Jinliang Du, Haojun Zhu, Jiancao Gao, Gangchun Xu, Pao Xu

**Affiliations:** 1 Wuxi Fisheries College, Nanjing Agricultural University, Wuxi 214081, China; zhongcy@ffrc.cn (C.Z.);; 2 Key Laboratory of Freshwater Fisheries and Germplasm Resources Utilization, Ministry of Agriculture and Rural Affairs, Freshwater Fisheries Research Center, Chinese Academy of Fishery Sciences, Wuxi 214081, China; zhuhaojun@ffrc.cn (H.Z.);; 3 International Joint Research Laboratory for Fish Immunopharmacology, Freshwater Fisheries Research Center, Chinese Academy of Fishery Sciences, Wuxi 214081, China

**Keywords:** Chinese mitten crab (*Eriocheir sinensis*), intestinal tissue, deltamethrin, oxidative stress, intestinal microbiome

## Abstract

The intestine is an important immune organ of aquatic animals and it plays an essential role in maintaining body health and anti-oxidative stress. To investigate the toxic effects of deltamethrin in intestinal tissue of Chinese mitten crabs (*Eriocheir sinensis*), 120 healthy crabs were randomly divided into two experimental groups (blank control group and deltamethrin-treated group), with three replicates in each group. After being treated with deltamethrin for 24 h, 48 h, 72 h, and 96 h, intestinal tissues were collected aseptically to assess the effects of deltamethrin on oxidative stress, immunity, apoptosis-related genes, and the structure of microflora in intestinal tissues. Additionally, correlations between gut microbiota composition and intestinal tissue damage-associated genes were analyzed. The results demonstrated that prolonged exposure to deltamethrin induced oxidative stress damage in intestinal tissue. Compared with the blank control group, the expression of autophagy-related genes B-cell lymphoma/Leukemia-2 (*bcl-2*), c-Jun N-terminal kinase (*jnk*), Microtuble-associated protein light chain 3 (lc3c), Cysteine-dependent Aspartate-specific Protease 8 (caspase 8), BECN1(beclin1), oxidative stress damage-related genes MAS1 proto-oncogene (mas), Glutathione Peroxidase (*gpx*), kelch-like ECH-associated protein 1 (keap1), Sequestosome 1 (*p62*), Interleukin-6 (*il-6*), and immune-related genes Lipopolysaccharide-induced TNF-alpha Factor (*litaf*), Heat shock protein 90 (hsp90) and prophenoloxidase (propo) in the deltamethrin treatment group were significantly up-regulated at 96 h (*p* < 0.05 or *p* < 0.01). Additionally, 16S rRNA sequencing showed that the diversity of intestinal flora in the deltamethrin-treated group was significantly higher compared with the blank control group (*p* < 0.01). Analysis of the differences in the composition of intestinal flora at the genus level showed that the relative abundance of *Candidatus Bacilloplasma* in the deltamethrin treatment group was significantly lower than that in the blank control group (*p* < 0.01). In contrast, the relative abundances of *Flavobacterium*, *Lachnospiraceae_NK4A136_group*, *Acinetobacter*, *Chryseobacterium*, *Lacihabitans*, *Taibaiella*, *Hydrogenophaga*, *Acidovorax*, and *Undibacterium* were significantly higher than those in the blank control group (*p* < 0.05 or *p* < 0.01). Pearson correlation analysis revealed that *Malaciobacter*, *Shewanella*, and *Prevotella* exhibited significant positive correlations with gene indicators (*jnk*, *gpx*, lc3c, *litaf*, hsp90), while *Dysgonomonas*, *Vibrio*, and *Flavobacterium* demonstrated significant negative correlations with multiple gene indicators (caspase 8, p62, *il-16*, keap1, *jnk*, etc). These results demonstrate that deltamethrin significantly impacts the gut microbiota, immune function, and antioxidant capacity of *E. sinensis*. The changes in gut microbiota have correlations with the biomarkers of intestinal tissue injury genes, indicating that gut microbiota plays a crucial role in deltamethrin-induced intestinal tissue damage. These insights contribute to a better understanding of the ecological risks associated with deltamethrin exposure in aquatic organisms.

## 1. Introduction

River crab, also known as Chinese mitten crab (*Eriocheir sinensis*), is an important breeding variety in China and well received by consumers. In 2023, the total national output reached 888,600 tons. From 2014 to 2016, a serious outbreak of Hepatopancreatic necrosis disease (HPND) of *E. sinensis* occurred in Xinghua City, Jiangsu Province, which caused huge economic losses to farmers. After dissection, the crab showed empty shell and fluid accumulation, atrophied hepatopancreas, grayish-yellow or grayish-white, no food residue in the intestine, and some of the diseased crabs had yellowish-white contents in the intestine. The disease also exists in other areas; for example, in June 2022, more than 80% of the crabs in a farm in Kunshan showed symptoms of “HPND” [1]. In 2024, a suspected death of *E. sinensis* due to “HPND” occurred in a river crab farm in Liyang City, Jiangsu Province [2]. There are various opinions regarding the etiology of the disease. Some scholars attributed disease occurrence to deltamethrin [3], while others propose that microsporidium is the primary factor [4]. However, the specific pathogenesis of the disease has not yet been concluded.

Deltamethrin, a pyrethroid broad-spectrum insecticide, is extensively employed in agricultural production for its effectiveness in controlling various pests. In aquaculture, deltamethrin is used extensively aquaculture, mainly for controlling parasitic infections in fish, and is also applied for pond cleaning after crab culture to remove zooplankton such as rotifers, cladicornis, and tickopods. Studies have reported that deltamethrin has a half-life of 10.465 days in water, 104.321 days in soil, and 11–15 days in the plasma and hepatopancreas of *E. sinensis* within a simulated rice field culture system [5]. Investigations into deltamethrin’s acute toxicity on aquatic organisms have been conducted by several researchers. Han et al. demonstrated that deltamethrin induces neurotoxic effects in *Penaeus vannamei*, manifesting as erratic swimming, wall-bumping, and convulsions [6]. Chen et al. reported that deltamethrin can cause biochemical toxicity in tilapia by inhibiting catalase (CAT) activity while inducing and activating monoamine oxidase (MAO) content in the liver and muscle tissue of Blue tilapia (*Oreochromis aureus*) [7]. Long-term exposure to deltamethrin has been shown to induce inflammatory responses, oxidative stress, and genotoxic effects in common carp (*Cyprinus carpio*), as reported by Arslan et al. [8]. As benthic organisms, crabs exhibit heightened susceptibility to pyrethroid pesticides. It has been reported that deltamethrin was enriched in all tissues of *E. sinensis*, and the content of deltamethrin in each tissue reached a peak after 0.5 h [9]. Therefore, it is worth investigating the potential toxicity of deltamethrin in *E. sinensis* to assess ecological safety in both aquaculture practices and aquatic ecosystems.

At present, there are few studies on the toxic mechanism of deltamethrin to aquatic animals. Liu et al. believed that deltamethrin primarily affects *Odontobutis potamophila* by inhibiting gill tissue energy metabolism and liver antioxidant capacity, which disrupts normal physiological functions and induces toxic responses [10]. Further studies on *E. sinensis* revealed significant hepatopancreatic damage caused by deltamethrin exposure. Yang et al. found that deltamethrin caused obvious damage to hepatopancreas of *E. sinensis*. And the malondialdehyde (MDA) levels in the hepatopancreas was significantly increased, alongside fluctuating superoxide dismutase (SOD) and catalase (CAT) enzyme activities, which are signs of oxidative stress [11]. Immunotoxic effects of deltamethrin have also been observed in other aquatic species. Zhang found that exposure to deltamethrin reduced immune factor levels in *Gobiocypris rarus* and impaired their immune response [12]. Similarly, Chen et al. found that deltamethrin could significantly inhibit the activity of AchE in tilapia serum, with an inhibition rate of up to 62.3% [13]. Ren et al. provided evidence that deltamethrin could cause neurotoxicity to zebrafish, as evidenced by abnormal swimming behaviors [14]. Furthermore, Siwicki et al. reported that rainbow trout exposed to deltamethrin for 30 min showed a concentration-dependent decrease in respiratory burst and lysozyme activity, along with significant decreases in immunoglobulin IgM levels [15]. Despite these findings across various aquatic species, there remains a paucity of research specifically addressing the toxicological effects of deltamethrin on *E. sinensis*.

The intestine not only serves as a crucial site for nutrient digestion and absorption in aquatic animals but also plays a vital role in immune regulation under external stimulation. As the first barrier of defense in the digestive system, the intestinal tract plays a role in differentiating substances within the intestinal lumen and preventing the invasion of pathogenic antigens. A large number of microorganisms reside in the intestines of animals, and the balance of gut microbiota is essential for maintaining intestinal health. Once the balance is disrupted, opportunistic pathogens will invade various organs, ultimately leading to the outbreak of bacterial diseases [16]. Intestinal injury is accompanied by the destruction of intestinal structural integrity and a significant increase in intestinal barrier permeability, which induces ion dysregulation and inflammatory responses [17]. Some scholars put forward the theory of the gut–liver axis, which believes that there is a dynamic equilibrium between intestinal and liver function and that the gut and liver affect each other. Once the equilibrium is broken, the body will have an inflammatory reaction, resulting in body tissue damage, and, in severe cases, fatalities [18,19]. Current research on deltamethrin’s impact on gut microbiota has predominantly focused on insects and fish, which can reduce the relative abundance of beneficial bacteria and increase the relative abundance of pathogenic bacteria [20,21]. Zhang et al. investigated the effects of deltamethrin on the intestinal tissue of *E. sinensis* through histopathological section observation. The study revealed that deltamethrin exposure induced vacuolation and partial necrosis/exfoliation of the intestinal mucosal columnar epithelium in the crabs [22]. However, there are few reports on the mechanism of intestinal toxicity of deltamethrin in *E. sinensis*. Therefore, it is of great significance to study the mechanism of deltamethrin toxicity to the intestinal tissues of *E. sinensis* for harvest production and water environment protection.

In this study, juvenile *E. sinensis* specimens were selected as experimental subjects to systematically assess the toxicological effects of deltamethrin on intestinal tissue. By employing a comprehensive approach combining microbiology and molecular biological indicators, this study aims to enhance our understanding of the underlying mechanisms of deltamethrin and lay the foundation for the prevention and control of deltamethrin damage in aquaculture.

## 2. Materials and Methods

### 2.1. Experimental Material

#### 2.1.1. Experimental Crab

*Eriocheir sinensis* was purchased from Yangzhong Breeding Base of Freshwater Fisheries Research Center, Chinese Academy of Fisheries Sciences. All crabs were healthy and uninjured, with an approximate body weight of 11.0 ± 1.3 g. After being retrieved from the farm, the crabs were raised in the circulating water system for 2 weeks prior to the subsequent experimental operation. The water parameters in the circulating system were set as follows: water temperature: 23 ± 1 °C; pH: 7.2 ± 0.2; dissolved oxygen: >6 mg/L. The crabs were fed two times a day with commercial feed (Jiangsu Haipalui Feed Co., Ltd., Taizhou, China). The crabs fasted for 24 h before the deltamethrin exposure experiments, and feeding and water changes were prohibited throughout the trial period.

#### 2.1.2. Chemicals and Reagents

Deltamethrin was purchased from Sigma Aldrich (Shanghai) Trading Co., Ltd. (CAS: 52918-63-5, HPLC ≥ 95%) (Shanghai, China). Reverse transcription kits and quantitative real-time PCR kits were purchased from Takara Biomedical Technology (Beijing) Co., Ltd. (Beijing, China).

### 2.2. Experimental Design

A total of 120 healthy *E. sinensis* were randomly divided into two experimental groups (60 in each group) and named as the blank control group and the deltamethrin treatment group. Each group included 60 individuals with 3 replicates. The study’s trial protocol was delineated as follows: No deltamethrin was added in the blank control group. The experimental group was administered deltamethrin at a concentration of 4.317 μg/L, equivalent to 60% of the determined LC50, according to our previous study [23]. Five crabs per replicate were randomly selected for subsequent trials. Intestinal tissues of *E. sinensis* were collected 24 h, 48 h, 72 h, and 96 h after freezing anesthesia. All procedures were performed under sterile conditions, and the samples were stored in a −80 °C freezer. All experimental protocols were performed in strict adherence to the Laboratory’s Animal Management Guidelines and were ethically reviewed and approved by the Institutional Animal Care and Use Committee of Freshwater Fisheries Research Center, Chinese Academy of Fishery Sciences (Permit No. LAECFFRC-2024-06-15).

### 2.3. 16S rRNA Gene Diversity Sequencing of Eriocheir sinensis

In this study, we collected 48 h intestinal tissue samples for microbial diversity detection, and 6 intestinal tissue samples were collected from each group for detection. A DNA extraction kit was used to extract DNA from the samples. The quality and concentration of the DNA extracted from the samples were determined using a NanoDrop 2000 spectrophotometer (Thermo Fisher Scientific, Waltham, MA, USA) and agarose gel electrophoresis. PCR amplification of the V3-V4 hypervariable regions of the bacterial 16S rRNA gene was carried out in a 25 μL reaction using universal primer pairs (343F: 5′-TACGGRAGGCAGCAG-3′; 798R: 5′-AGGGTATCTAATCCT-3′) [24]. Agarose gel electrophoresis was used to assay the PCR amplification products obtained from the first-round amplification. The purified products were amplified by secondary PCR, and the obtained products were tested by electrophoresis again. The purified products were quantitatively detected by Qubit method after purification with magnetic beads, and the same amount of mixed samples was analyzed according to the concentration of PCR products. The sequencing was conducted by Shanghai OE Biotechnology Co., Ltd. using the Illumina NovaSeq 6000 sequencing platform (Illumina Inc., San Diego, CA; OE Biotech Company; Shanghai, China).

### 2.4. Real-Time Quantitative RT-PCR

Total RNA was extracted from the intestinal tissues of each experimental group according to the instructions of the TaKaRa MiniBEST Universal RNA Extraction Kit. Quality assessment of RNA extraction was performed using a NanoDrop 2000 spectrophotometer (Thermo Fisher Scientific, Waltham, MA, USA). After the determination, reverse transcription was performed to synthesize the RNA into cDNA. Quantitative PCR amplifications were carried out using the CFX96 Real-time PCR Detection System (Bio-Rad Laboratories, Inc., Hercules, CA, USA) according to the TB Green™ SYBR Premix Ex Taq™ manual. The quantitative primer sequences of target genes and reference gene β-actin are presented in Table 1. Gene expression quantification was calculated by the 2^−∆∆Ct^ method [25].

### 2.5. Statistical Analysis

After verifying the normality of the data using the Shapiro–Wilk test and homogeneity of variance with Levene’s test in the SPSS 27.0 software package, the *t*-test was used to analyze the significance of differences between the two different treatment groups at the same time point. The experimental data were expressed as mean ± standard error (SEM), and *p* < 0.05 was considered to indicate significant differences.

Data analysis of alpha and beta diversity of gut microbes was performed using QIIME 2 software. The alpha diversity indices included the Chao1 index, ACE index, Shannon index, Simpson index, etc. An alpha diversity boxplot was used to analyze and calculate the difference significance of the diversity index in different treatment groups. The beta diversity of samples was evaluated by PCoA analysis based on the Bray–Curtis distance matrix algorithm, combined with the difference significance *p*-value in Adonis and Anosim analysis. Based on the relative abundance of species among samples, spearman correlation coefficient was calculated to obtain the correlation network map between species within the sample. The Pearson calculation method was used for correlation analysis, and a threshold of correlation coefficient ≥ 0.8 and *p* ≤ 0.05 were considered as the criteria for significant correlation.

## 3. Results

### 3.1. Sequencing Data

**Figure 1:**
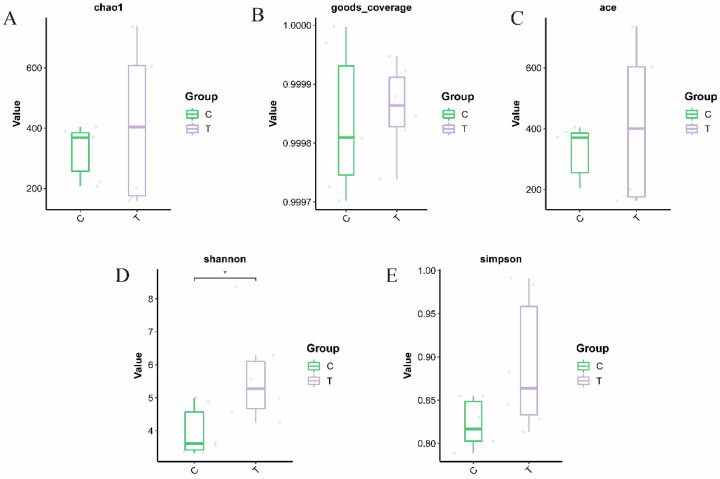
Effects of deltamethrin on alpha diversity indices—Chao (**A**), goods_coverage (**B**), ACE (**C**), Shannon (**D**), and Simpson (**E**)—of intestinal microbes in *E. sinensis*. C: control. T: treatment for 48 h with deltamethrin. The asterisk “*” indicates significant differences between the control and deltamethrin treatment groups (*p* < 0.05).

**Figure 2:**
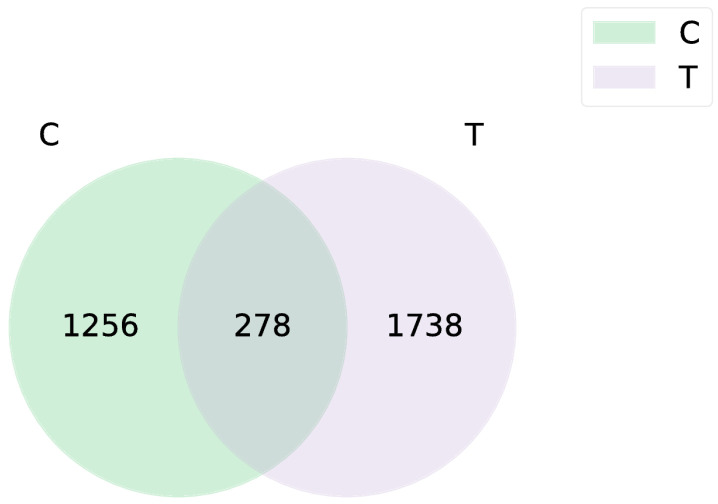
Venn diagram showing the distribution of crabs sharing ASVs after exposure to deltamethrin for 48 h. The numbers represent the relevant ASVs in each group of total sequences. C: control. T: treatment for 48 h with deltamethrin.

**Figure 3:**
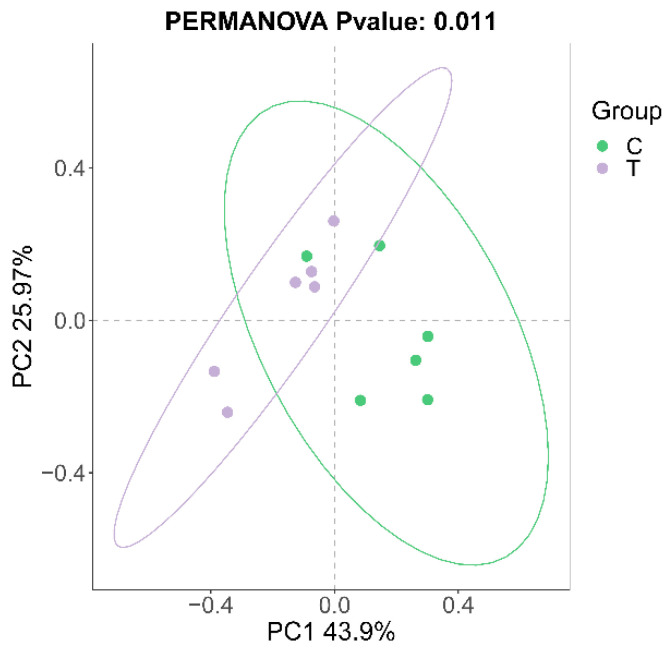
Effects of deltamethrin on the beta diversity index of intestinal microbes in *E. sinensis*. C: control. T: treatment for 48 h with deltamethrin.

A total of 744,250 valid sequences and 4435 cluster units (amplicon sequence variants, ASVs) were obtained by 16S rRNA high-throughput sequencing, belonging to 31 phyla, 65 classes, 156 orders, 260 families, 443 genera, and 666 species. The alpha diversity indices (ACE, Chao, Shannon, Simpson) of the blank control group were significantly lower compared to the deltamethrin treatment group (Figure 1). Notably, the Shannon index in the deltamethrin treatment group was significantly higher relative to the blank control group (*p* < 0.05). A total of 278 ASVs were found between the control group and the deltamethrin treatment group, while 1256 and 1738 ASVs were found in the blank control and deltamethrin treatment groups, respectively, showing a substantial difference between the two groups (Figure 2). In addition, principal coordinate analysis (PCoA) results (Figure 3) revealed significant differences in the composition of intestinal flora between the blank control group and the deltamethrin treatment group (*p* < 0.05).

### 3.2. Comparative Analysis of Gut Microbial Community Composition Between Two Groups of Eriocheir sinensis at the Phylum Level

**Figure 4:**
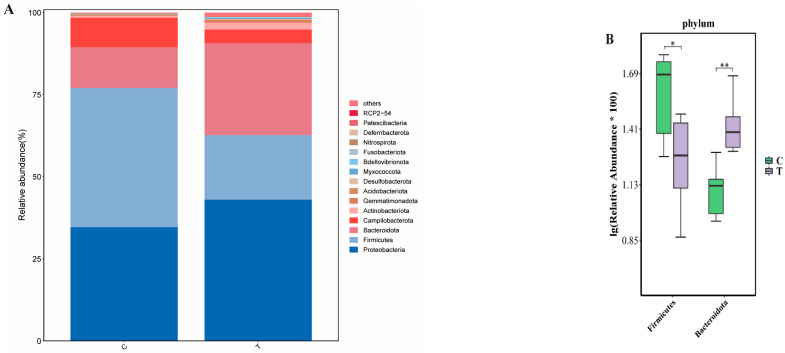
Comparison of the differences in the structure (**A**) and composition of intestinal microflora (**B**) of *E. sinensis* between the blank control group and the deltamethrin exposure group at the phylum level. C: control. T: treatment for 48 h with deltamethrin. The asterisk “*” “**”indicates significant differences between the control and deltamethrin treatment groups (*p* < 0.05 or *p* < 0.01).

The results of species composition analysis (Figure 4) showed that the dominant intestinal flora in both the blank control group and the deltamethrin treatment group were Proteobacteria, Firmicutes, Bacteroidetes, and Campilobacterota. The relative abundance of Firmicutes and Bacteroidetes in the two treatment groups was significantly different from those in the blank control group (*p* < 0.05 or *p* < 0.01).

### 3.3. Comparative Analysis of Gut Microbial Community Composition Between Two Groups of Eriocheir sinensis at the Genus Level

**Figure 5:**
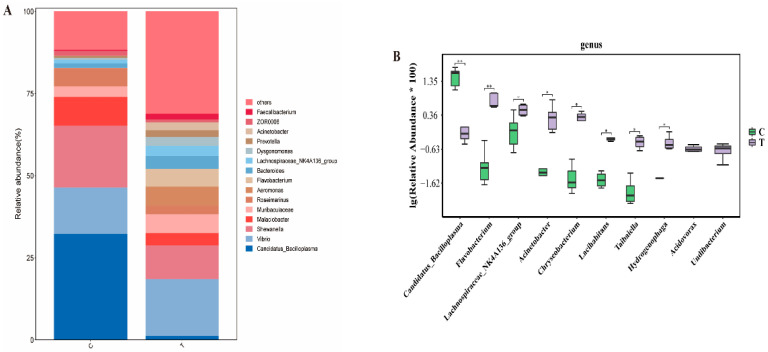
Comparison of the differences in the structure (**A**) and composition of intestinal microflora (**B**) of *E. sinensis* between the blank control group and the deltamethrin exposure group at the genus level. C: control. T: treatment for 48 h with deltamethrin. The asterisk “*” “**”indicates significant differences between the control and deltamethrin treatment groups (*p* < 0.05 or *p* < 0.01).

At the genus level, the dominant bacteria genera in the blank control group and the deltamethrin treatment group were *Candidatus Bacilloplasma*, *Vibrio*, *Shewanella*, *Malaciobacter*, *Muribaculaceae*, *Roseimarinus*, *Aeromonas*, *Flavobacterium*, *Bacteroides*, *Lachnospiraceae_NK4A136_group*, *Dysgonomonas*, *Prevotella*, *Acinetobacter*, *ZOR0006*, *Faecalibacterium*, and other bacteria (Figure 5A). After deltamethrin exposure, the relative abundance of *Candidatus Bacilloplasma* in the deltamethrin-treated group was significantly lower than that in the blank control group (*p* < 0.01); In contrast, the relative abundance of *Flavobacterium*, *Lachnospiraceae_NK4A136_group*, *Acinetobacter*, *Chryseobacterium*, *Lacihabitans*, *Taibaiella*, *Hydrogenophaga*, *Acidovorax*, and *Undibacterium* was significantly higher compared with the blank control group (*p* < 0.05 or *p* < 0.01) (Figure 5B).

### 3.4. LEfSe Species Analysis

**Figure 6:**
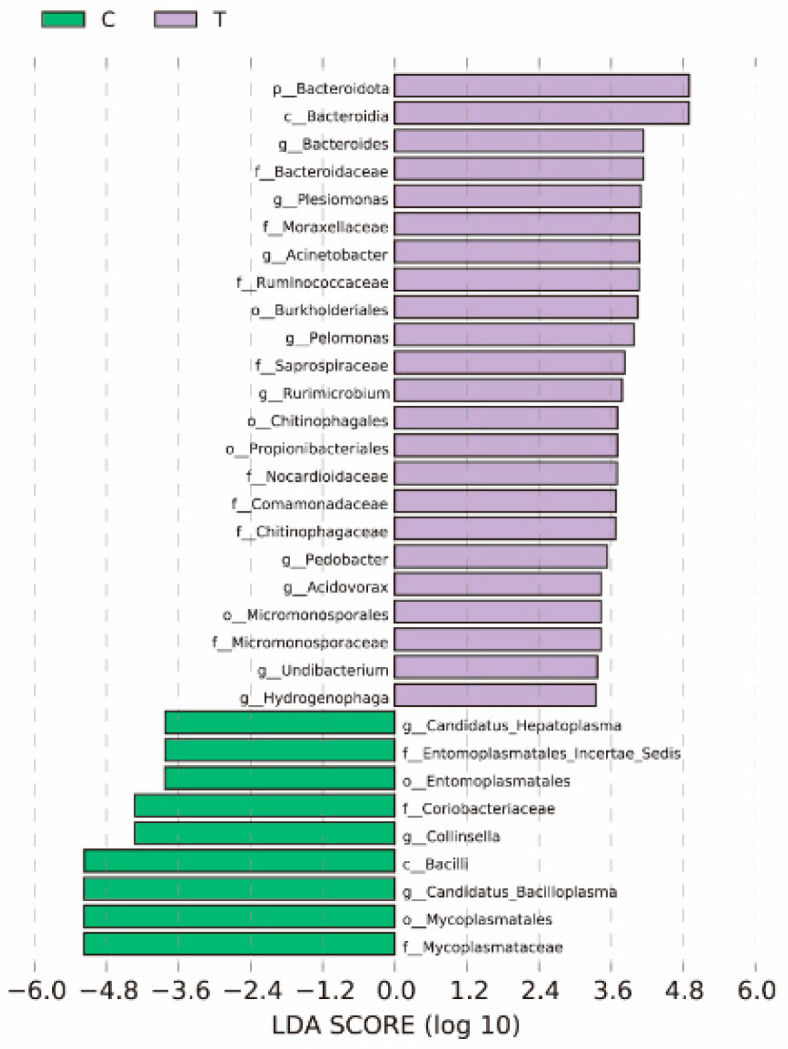
Bar chart for comparison of different species of intestinal microbes in *E. sinensis* between the blank control group and deltamethrin exposure group. Different colors indicate different groups: light purple bars indicate species with relatively high abundance in deltamethrin exposed groups, and light green bars indicate species with relatively high abundance in blank control groups. C: control. T: treatment for 48 h with deltamethrin.

As shown in Figure 6, a total of 32 marker species with significant group differences were selected after filtering by LEfSe analysis. There were nine marker species in the blank group. Among them, the differential markers at the genus level are *Candidatus Arthromitus*, *Collinsella*, and *Candidatus Bacilloplasma*. There were 23 markers in the deltamethrin treatment group. Among them, the differential markers at genus level are *Bacteroides*, *Pesiomonis*, *Acnetoacte*, *Peamonas*, *Rurimicrobkmn*, *Pedobscter*, *Acdovorax*, *Hydrogenpphag*, and *Undibacterium*.

### 3.5. Correlation Analysis of Microbiota

**Figure 7:**
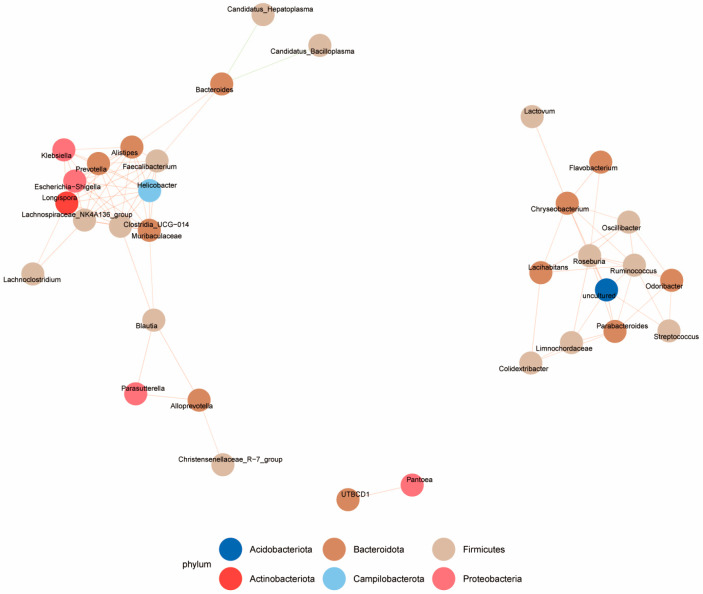
Diagram of the interrelationship network between the two groups of species. Different colors are used to denote different species. The color of the line indicates positive and negative correlation: red represents a positive correlation, while green represents a negative correlation. The thickness of the line corresponds to the magnitude of the Pearson correlation coefficient. Specifically, as the line gets thicker, the correlation between species becomes stronger. In addition, a species with a larger number of lines has a closer relationship with other species.

Spearman rank correlation coefficient was used to analyze the relative abundances of the top 50 genera in the control group and the deltamethrin treatment group, respectively. The correlation between the dominant genus was obtained based on |SpearmanCoef| > 0.8 and *p* < 0.01, which facilitated the construction of the association network (Figure 7). The results indicated that Bacteroides had a negative correlation with *Candidatus Bacilloplasma* and *Candidatus Hepatoplasma* while showing a positive correlation with *Faecalibacterium*, *Alistipes*, *Klebsiella*, *Prevotella*, *Escherichia-Shigella*, *Lachnospiraceae_NK4A136_group*, *Helicobacter*, *Clostr idia_UCG-014*, and *Lachnoclostridium*. Additionally, UTBCD1 had a positive correlation with *Pantoea*. *Chryseobacterium* had a positive correlation with *Flavobacterium*, *Roseburia*, *Oscillibacter*, *Lacihabitans*, *Ruminococcus*, *Odoribacter*, *Streptococcus*, *Parabacteroides*, *Limnochordaceae*, and *Colidextribacter*.

### 3.6. Functional Predictive Analysis

**Figure 8:**
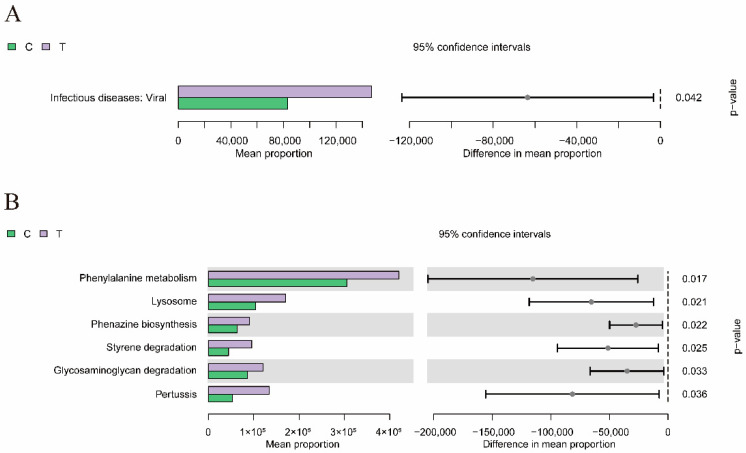
Bar plots of the difference analysis of intestinal microbial genes in KEGG metabolic pathways at levels 2 (**A**) and 3 (**B**). C: control. T: treatment for 48 h with deltamethrin.

As shown in Figure 8, at KEGG level 2, there were statistically significant differences in categories related to infectious diseases: Viral (*p* < 0.05). Furthermore, at the KEGG level 3, there were significant differences in Amoebiasis, Prolactin signaling pathway, Lysosome, Apoptosis, N_Glycan biosynthesis, and Carotenoid biosynthesis, MicroRNAs in cancer, Retinol metabolism, Phenylalanine metabolism, Phenazine biosynthesis, Styrene degradation, MAPK signaling pathway-yeast, Pertussis, Tryptophan metabolism, Glycosaminoglycan degradation, Phenylalanine metabolism, Lysosome, Phenazine biosynthesis, Styrene degradation, Glycosaminoglycan degradation, and Pertussis (*p* < 0.05).

### 3.7. Analysis of Phenotypic Differences Among Different Groups of Microorganisms

**Figure 9:**
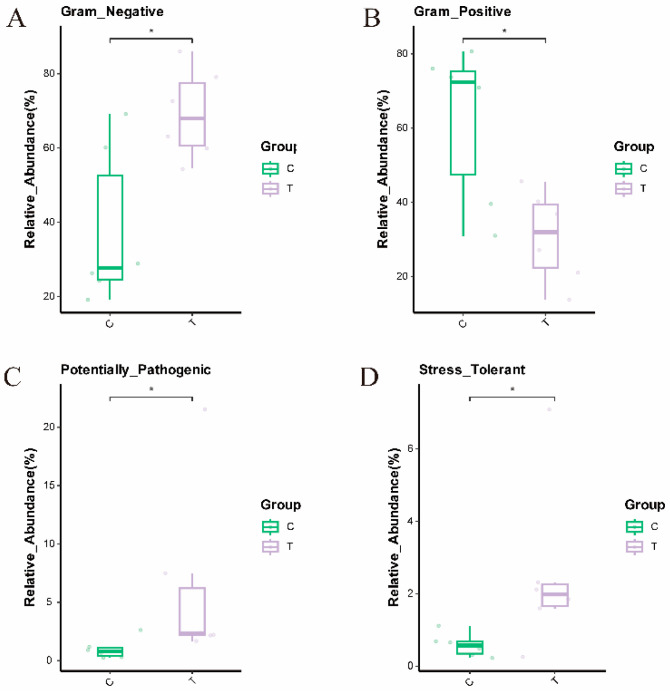
Analysis diagram of functional difference results of Gram-negative bacteria (**A**), Gram-positive bacterial (**B**), Potential pathogenicity (**C**), and Stress tolerance (**D**) of different treatment groups. The dates are presented as mean ± SEM (*n* = 6). C: control. T: treatment for 48 h with deltamethrin. The asterisk “*” indicates significant differences between the control and deltamethrin treatment groups (*p* < 0.05).

As shown in Figure 9, after phenotypic analysis of microbial data for the blank control group and the deltamethrin treatment group using the Bugbase tool, it was found that the relative abundance of Gram-negative bacteria, potential pathogenicity, and oxidative stress tolerance in the deltamethrin treatment group increased significantly after 48 h of deltamethrin treatment (*p* < 0.05); the abundance of Gram-positive bacterial communities was significantly reduced (*p* < 0.05).

### 3.8. Effects of Deltamethrin on Apoptosis and Autophagy-Related Genes in Intestinal Tissues

**Figure 10:**
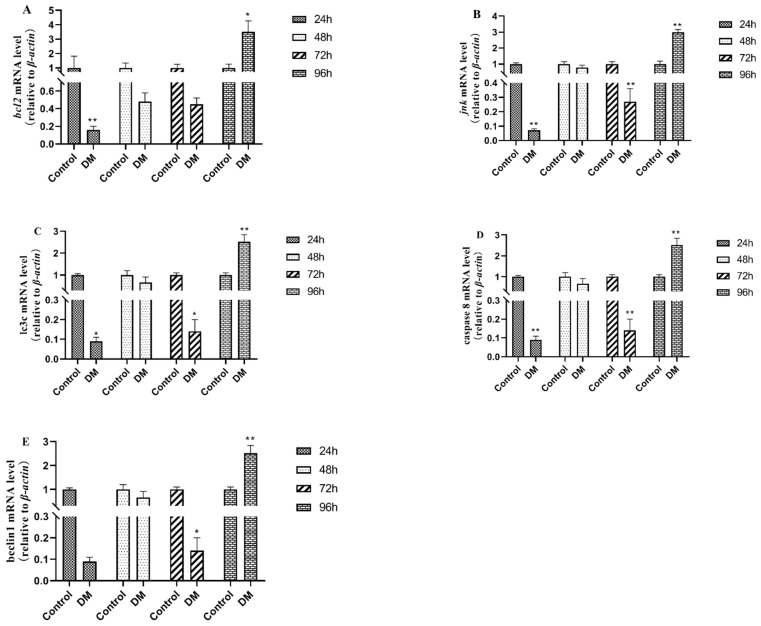
Effects of deltamethrin on the mRNA expression of *bcl-2* (**A**), *jnk* (**B**), *lc3c* (**C**), *caspase 8* (**D**), and beclin1 (**E**) in intestinal tissue of *E. sinensis*. The dates are presented as mean ± SEM (*n* = 6). The asterisk “*” “**”indicates significant differences between the control and deltamethrin treatment groups (*p* < 0.05 or *p* < 0.01).

As shown in Figure 10, the gene expressions of *bcl-2*, *jnk*, lc3c, caspase 8, and beclin1 in intestinal tissues were all down-regulated after 24 h, 48 h, and 72 h and up-regulated 96 h after deltamethrin exposure, which were significantly different from those in the blank control group (*p* < 0.05 or *p* < 0.01).

### 3.9. Effects of Deltamethrin on Genes Related to Oxidative Stress Injury in Intestinal Tissue

**Figure 11:**
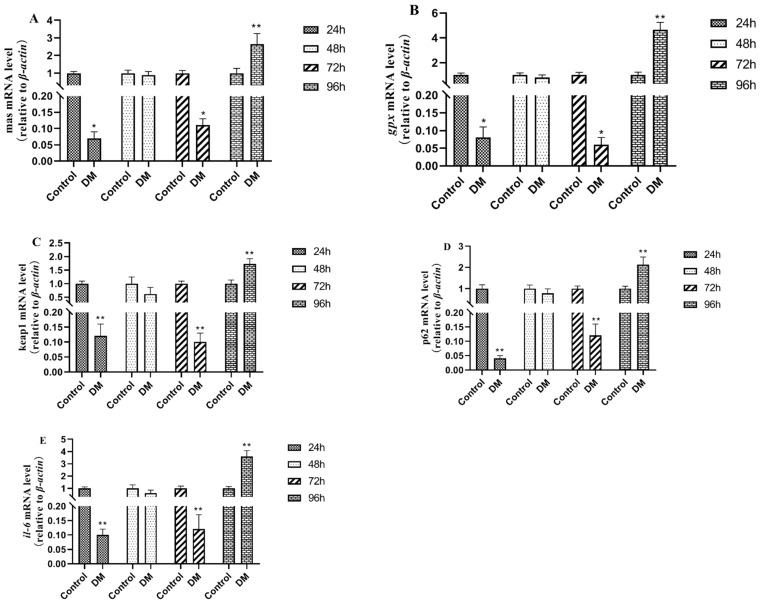
Effects of deltamethrin on the mRNA expression of mas (**A**), *gpx* (**B**), keap1 (**C**), p62 (**D**), and *il-6* (**E**) in intestinal tissue of *E. sinensis*. The dates are presented as mean ± SEM (*n* = 6). The asterisk “*” “**” indicates significant differences between the control and deltamethrin treatment groups (*p* < 0.05 or *p* < 0.01).

As shown in Figure 11, the expressions of the mas, *gpx*, keap1, p62, and *il-6* genes related to oxidative stress damage in intestinal tissues after deltamethrin exposure were all down-regulated after 24 h, 48 h, and 72 h and up-regulated after 96 h, with significant differences compared with the blank control group (*p* < 0.01).

### 3.10. Effects of Deltamethrin on Immune-Related Genes in Intestinal Tissues

**Figure 12:**
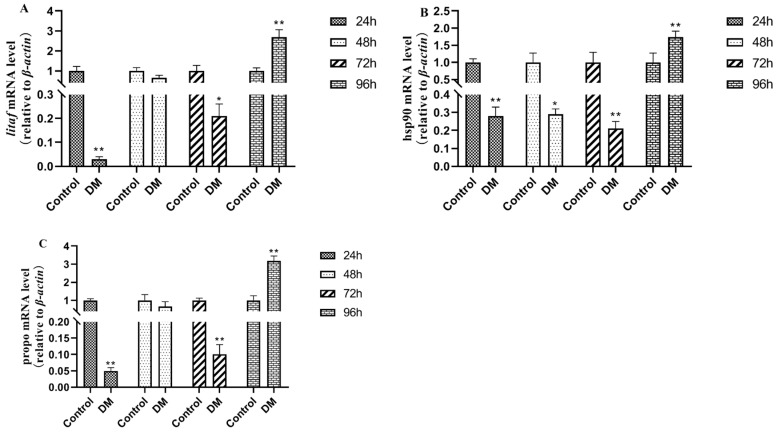
Effects of deltamethrin on the mRNA expression of *litaf* (**A**), hsp90 (**B**), and propo (**C**) in intestinal tissue of *E. sinensis*. The data are presented as mean ± SEM (*n* = 6). The asterisk “*” “**” indicates significant differences between the control and deltamethrin treatment groups (*p* < 0.05 or *p* < 0.01).

As shown in Figure 12, after deltamethrin exposure, the expressions of immune-related genes *litaf*, hsp90, and propo in intestinal tissues showed a down-regulated trend after 24 h, 48 h, and 72 h, as well as an up-regulated trend after 96 h, with significant differences relative to the blank control group (*p* < 0.01).

### 3.11. Correlation Analysis of Intestinal Microbiota and Intestinal Tissue Damage Genes

**Figure 13:**
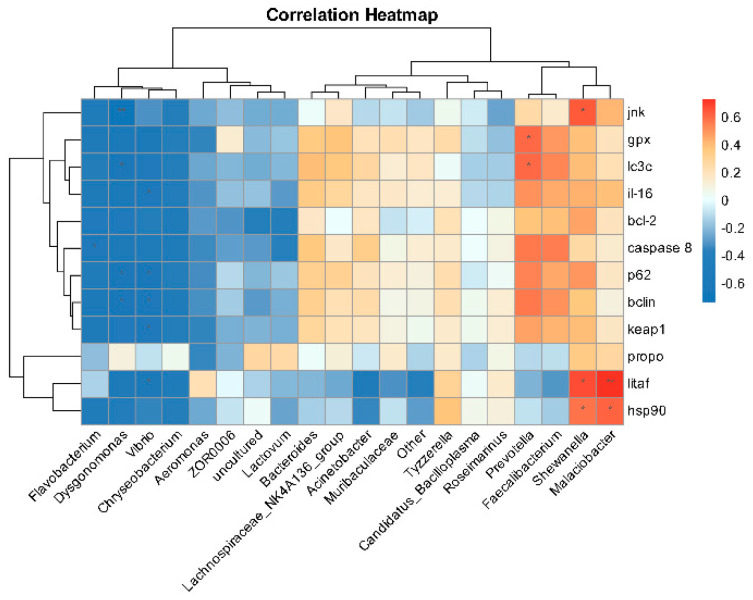
Clustered heatmap of the correlation between gut microbiota and gene expression. The asterisk “*” “**” indicates significant differences between intestinal flora and intestinal tissue damage genes (*p* < 0.05 or *p* < 0.01).

As shown in Figure 13, the Pearson correlation calculation method was conducted to investigate the impact of deltamethrin exposure on the relationships between gene expression-related indicators and specific intestinal microbiota in *E. sinensis*. The results indicated that *Malaciobacter*, *Shewanella*, and *Prevotella* exhibited significant positive correlations with gene indicators (*jnk*, *gpx*, lc3c, *litaf*, hsp90). In contrast, *Dysgonomonas*, *Vibrio*, and *Flavobacterium* demonstrated significant negative correlations with multiple gene indicators (caspase 8, p62, *il-16*, keap1, *jnk*, etc).

## 4. Discussion

Numerous domestical and international scholars have conducted systematic studies on the structure of the intestinal tissue in crustaceans. The digestive system of crustaceans is composed of three primary segments: the foregut, midgut, and hindgut [27,28]. Among these segments, the midgut represents the most critical component of the digestive system, responsible for both enzymatic secretion and nutrient absorption. The gut-associated immune system in crustaceans constitutes a crucial protective mechanism against external pathogens, comprising three essential barriers: the mechanical barrier, biological barrier, and immune barrier. These three barriers play an important role in body health defense [29,30]. In our previous study, we set three concentration groups of 20%-96 h LC50, 40%-96 h LC50, and 60%-96 h LC50 (1.439 μg/L, 2.878 μg/L, and 4.317 μg/L) to study the toxic effects of deltamethrin on *E. sinensis*. Comprehensive analyses were conducted on immune parameters, antioxidant defense systems, and lipid metabolism profiles in both serum and hepatopancreas tissues. Comparison of the results of enzyme activities measured at these three concentrations, all enzyme activity levels in the DM-4.317 μg/L group were significantly different with time variation. Notably, the 4.317 μg/L exposure group (equivalent to 60% of 96-h LC50) induced significant hepatopancreatic injury after 48 h of exposure [23]. The aim of this study is to investigate the toxicological effects of deltamethrin on the intestinal tissue of *E. sinensis*, focusing specifically on its impacts on the biological and immune barriers.

B-cell lymphoma/Leukemia-2 (*bcl2*) is an important apoptosis suppressor gene, mainly located in the cytoplasmic surface of the nuclear membrane, endoplasmic reticulum, and mitochondrial outer membrane, which can inhibit apoptosis caused by lipid membrane peroxidation and ionizing radiation and form a dimer itself or a heterodimer with proteins such as Bax to exert its function of regulating apoptosis. Wang et al. observed a significant up-regulation of *bcl2* protein expression levels in injured mouse brain cells induced by bacterial lipopolysaccharides [31]. Li indicated that alcohol can induce up-regulation of the gene *bcl2* in the injured liver tissue of zebrafish [32]. Zhang et al. reported that acenaphthene caused liver toxicity in zebrafish, resulting in a significant up-regulation of *bcl2* [33]. As a pivotal component of the mitogen-activated protein kinase (MAPK), c-Jun N-terminal kinase (*jnk*) can be activated by various factors, including drugs, environmental factors, and pathogenic elements. Li found that aflatoxin B1 can induce liver injury and significantly up-regulate the expression of *jnk* and other genes related to endoplasmic reticulum stress in the liver of Snakehead fish [34]. Microtuble-associated protein light chain 3 (LC3) is a key member of the autophagy-related protein (ATG) family and plays a crucial role in the process of autophagy. Autophagy proteins regulate cellular metabolism and homeostasis, thereby participating in various biological processes [35]. Beclin is a recognized autophagy regulator in the autophagy protein family, which can interact with other proteins to participate in biological processes such as autophagy, apoptosis, and immune modulation [36]. The Cysteine-dependent Aspartate-specific Protease 8 (caspase 8) protein is a key mercaptan protease that acts as a molecular switch in apoptosis, inducing exogenous cell apoptosis through the cleavage and activation of effector caspase, and it is essential for preventing tissue damage [37]. The findings of this experiment are in accordance with previously published studies. In this study, the gene expression of *bcl-2*, *jnk*, lc3c, caspase 8, and beclin1 all showed a trend of first down-regulation and then up-regulation, indicating that the body first responded to deltamethrin exposure to resist the effects of deltamethrin, due to the continuous stimulation of deltamethrin, the body needs to initiate apoptosis procedures to eliminate damaged cells, leading to subsequent up-regulation of these genes, reflecting a response to potential cell damage or apoptosis processes.

Oxidative stress damage-related genes MAS1 proto-oncogene (mas) belongs to the G protein-coupled receptor family and plays an important role in various physiological processes. Recent studies have pointed out the involvement of the *mas* gene in acetaminophen-induced liver injury in mice, with significant up-regulation observed in mouse liver tissue [38]. The glutathione peroxidase (*gpx*) gene is not only known for its antioxidant effects in alleviating inflammatory damage but also directly regulates inflammatory signaling pathways. Its primary regulatory mechanism involves inhibiting NF-κB activation and reducing the production of inflammatory factors, thereby mitigating inflammatory injury. It has been found that the expression level of the *gpx* gene in the hepatopancreas of *Litopenaeus vannamei* was significantly up-regulated after being fed a diet containing chlorogenic acid for 28 days, indicating that the *gpx* gene plays an important role in the antioxidant system of *Litopenaeus vannamei* in response to low salinity stress [39]. In aquatic animals, the kelch-like ECH-associated protein 1 (keap1) gene is closely associated with injury responses. Normally, keap1 binds to the nuclear transcription factor *nrf2*, maintaining its inactive state. However, when aquatic animals are subjected to oxidative stress, a conformational change in keap1 reduces its binding affinity to *nrf2*, allowing *nrf2* to translocate into the nucleus, then activating the expression of a series of downstream antioxidant genes such as *gpx* and interleukin-6 (*il-6*), while also regulating immune cell functions and enhancing the organism’s immune defense capabilities [40]. As a multifunctional protein, Sequestosome 1(p62) is involved in the oxidative stress response of cells. Under circumstances such as oxidative stress, the level of p62 in cells will increase and promote autophagy to maintain cellular homeostasis [41]. In the present study, the gene expression of mas, *gpx*, keap1, p62, *and il-6* was significantly up-regulated in the hepatopancreas of the *E. sinensis* after being treated with deltamethrin for 96 h. These findings suggest that deltamethrin induces oxidative stress responses in the hepatopancreas, leading to cellular oxidative damage, and the activation of antioxidant gene expression cascades.

Immune-related genes Lipopolysaccharide-induced TNF-alpha Factor (*Litaf*), also known as lipopolysaccharide-induced tumor necrosis factor-α, plays a key role in mediating immune responses [42]. Upon stimulation by pathogen-associated molecular patterns such as lipopolysaccharide (LPS), *Litaf* will be activated, thereby promoting the production of inflammatory cytokines such as *tnf-α.* Heat shock protein 90 (hsp 90) is a highly conserved molecular protein that is widely present in various tissues, which plays a pivotal role in maintaining cellular homeostasis by binding to and stabilizing proteins damaged by oxidative stress, preventing their aggregation or degradation while facilitating repair or clearance [43]. When aquatic animals are stimulated by pathogens or exogenous factors, the immune system will be activated and produce immune responses, resulting in the up-regulation of *hsp90* gene expression [44]. Prophenoloxidase (*propo*) is an important enzyme in organisms, typically existing as an inactive precursor [45]. Upon the recognition of foreign stimuli, *propo* will be activated and play a central role of immune defense. In this study, after exposure to deltamethrin in water, the gene expressions of *litaf*, hsp90, and propo were first down-regulated and then up-regulated. The initial down-regulation may represent an adaptive response by the organism, potentially due to deltamethrin’s interference with transcriptional or translational processes, leading to reduced expression levels. Conversely, the subsequent up-regulation of these genes likely reflects the activation of the immune system in response to continuous exposure to deltamethrin, enabling the body to resist deltamethrin invasion.

The intestinal microbiota performs essential functions in the digestion and nutrient absorption processes of aquatic animals, which forms a critical immune defense system that prevents the invasion and colonization of pathogenic microorganisms, together with the intestinal epithelial barrier [46]. This study found that Proteobacteria, Bacteroidetes, Firmicutes, and Campylobacter were the dominant microflora in the intestinal of *E. sinensis*, which is different from the study from Hong et al. [47]. In this study, deltamethrin exposure significantly increased the relative abundance of Bacteroidetes and significantly reduced the relative abundance of Firmicutes. The results of this study are similar to those of previously published studies. Yang et al. found that prometryn could change the diversity and structure of gut microorganism communities in *E. sinensis* [48]. Shu et al. found that Proteobacteria, Bacteroidetes, and Firmicutes were confirmed to be involved in carbon cycling in the water environment [49]. Gibiino et al. reported that Bacteroidetes are involved in immune regulation and lipid metabolism, and a relative increase in the abundance of Bacteroidetes will increase the risk of disease [50]. Tsai et al. reported that Firmicutes/Bacteroidetes ratio is closely related to inflammatory bowel disease [51]. Changes in the intestinal microbiota structure in *E. sinensis* indicated that deltamethrin caused an imbalance of intestinal flora in *E. sinensis*, which may have adverse effects on its physiological state.

As a part of intestinal flora, intestinal candida community maintains the balance of intestinal microecology together with other intestinal flora. A reduction in *Candida* numbers may impair the intestine’s ability to resist pathogenic invasion, thereby increasing the risk of infection. Trojanowska et al. demonstrated that an imbalance in intestinal *Candida* can disrupt the mucosal barrier, triggering immune responses and inflammation [52]. Numerous studies have shown that *Flavobacterium* can adversely affect the health and growth of aquatic animals, leading to various diseases such as rotten gill disease, enteritis, septicemia, and other diseases in grass carp (*Ctenopharyngodon idella*) [53]. *Lachnospiraceae_NK4A136_group* belongs to Firmicutes and exists in the intestinal tissues of healthy individuals. Some scholars have reported that *Lachnospiraceae_NK4A136_group* is associated with intestinal diseases and metabolic diseases, and an imbalance of *Lachnospiraceae_NK4A136_group* flora in the intestine can lead to the pathogenesis of the body [54,55]. Furthermore, Wang et al. reported that a high-fat diet induces intestinal microbial metabolic abnormalities in mice, with a significant increase observed in the relative abundance of *Lachnospiraceae_NK4A136_group* [56]. *Acinetobacter* is a bacterial pathogen affecting a wide range of aquatic species, with the potential to cause various pathological conditions that not only compromise the health and growth of these organisms but also result in substantial economic losses for aquaculture operations [57,58,59]. Xue et al. found that the isolation of *Acinetobacter baumannii* from channel catfish caused them to exhibit intussusception and liver degeneration [60]. *Chryseobacterium* have been shown to cause significant damage to fish tissues and organs, ultimately leading to immune system compromise and mortality [61,62]. *Lacihabitans* and *Taibaiella* are widely distributed in the natural environment, but there are relatively few studies on these genera [63,64,65]. The genera *Bacteroides* and *Hydrogenophaga* are identified as the core microbiota in *Procambarus clarkii*, playing a crucial role in gastrointestinal metabolism by aiding in the organism’s ability to metabolize amino acids and carbohydrates [66]. *Acidovorax* is a Gram-negative bacterium belonging to the *Comamonadaceae* family. It is known as a pathogenic bacterium contributing to plant fruit spot disease and can also cause diseases in aquatic animals and septicemia in humans [67,68,69]. *Undibacterium* can decompose organic substances such as residual bait and feces in aquaculture environments and convert them into simple inorganic substances. This metabolic process helps reduce the accumulation of organic matter in water [70]. In this study, deltamethrin significantly reduced the relative abundance of *Candidatus Bacilloplasma* in the intestinal tissues of *E. sinensis* and significantly increased the relative abundance of *Flavobacterium*, *Lachnospiraceae_NK4A136_group*, *Acinetobacter*, *Chryseobacterium*, *Lacihabitans*, *Taibaiella*, *Hydrogenophaga*, *Acidovorax*, *Undibacterium*, indicating that deltamethrin caused microbial structure disorder. Our results are in accordance with a report from Jiao et al. (2021), who found that lambda-cyhalothrin polydopamine microcapsule suspension significantly affected the intestinal flora in *E. sinensis*. The relative abundances of *Candidatus_Bacilloplasma*, *Shewanella*, *Acinetobacter*, *Flavobacterium*, *Pseudomonas*, and *Aeromonas* were significantly increased, resulting in the imbalance of the microbiota [71]. These results suggest that deltamethrin exposure may lead to the enhanced susceptibility of *E. sinensis* to pathogen infections.

The analysis of Bugbase phenotypes in the microbial community of *E. sinensis* provides valuable insights for disease diagnosis and health assessment. By monitoring these phenotypic changes, the data serve as a critical foundation for early diagnosis, disease surveillance, and evaluating treatment effectiveness. In bugbase phenotype analysis, exposure to deltamethrin significantly enhanced the capacity of Gram-negative bacteria, pathogenicity, and oxidative stress in the microbial community, which gives us a hint about the involvement of gut microbiota in deltamethrin metabolism. Correlation analysis revealed the intricate relationships between gut microbiota and intestinal tissue injury. Auguste et al. demonstrated that Malaciobacter marinus can cause infection and death in bivalve larvae and adults [72]. *Arcobacter* is a zoonotic Gram-negative pathogen that widely exists in various water bodies and can cause gastroenteritis [73]. *Shewanella putrefaciens*, a member of *Shewanella*, which is the main bacterium affecting aquaculture, can cause the death of aquatic animals [74]. Larsen reported that *Prevotella* can activate inflammatory signaling pathways and promote the occurrence of intestinal inflammation-related diseases [75]. *Dysgonomonas*, which belongs to coccobacilli, is a type of facultative anaerobic bacteria. Sun et al. found that *Dysgonomonas* participated in the development of non-alcoholic fatty liver disease [76]. Vibrio species are significant pathogens in aquaculture, with *Vibrio anguillarum*, *Vibrio parahaemolyticus*, and *Vibrio alginolyticus* being the primary contributors to aquatic animal health issues. Notably, *Vibrio parahaemolyticus* is implicated in causing ascites and mortality in *E. sinensis* [77]. Opportunistic bacterial pathogens like *Flavobacterium* are notable, particularly *Flavobacterium columnare*, which induces severe tissue injuries to gills and skin in finfish [78,79]. In this study, correlation analysis showed that *Malaciobacter*, *Shewanella*, *Prevotella*, *Dysgonomonas*, *Vibrio*, and *Flavobacterium have obvious correlation with jnk*, *gpx*, *lc3c*, *litaf*, *hsp90*, etc., which suggests that immune dysfunction and inflammatory response induced by deltamethrin may be related to the imbalance of intestinal flora in *E. sinensis.* However, the mechanisms by which these intestinal bacteria exacerbate deltamethrin-induced intestinal tissue injury remain unclear, necessitating further research and validation.

## 5. Conclusions

In conclusion, molecular biological studies have found that deltamethrin causes oxidative stress damage, abnormal expression of immune-related genes, and dysfunction of intestinal microbial flora in *E. sinensis*. After deltamethrin treatment, the intestinal flora of *E. sinensis* was dominated by Gram-negative bacteria, which greatly increased the pathogenicity. There exists a certain degree of correlation between intestinal microbiota and genes associated with intestinal tissue injury. It is speculated that the structural change in the microbial flora may be one of the causes of body damage. Further studies are needed to clarify the association between microbial community and deltamethrin-induced intestinal tissue damage, with particular emphasis on exploring microbiota-mediated toxicity mechanisms (e.g., bacterial metabolite alterations, inflammatory responses, immune suppression, and endotoxin release pathways) as key directions for future research. The successful execution of this experiment laid a theoretical foundation for the further study on the toxicity of deltamethrin in crustaceans.

## Figures and Tables

**Table 1:** The primer sequences used for RT-PCR in the present study.

Type	Gene	Primer Sequence (5′-3′)	GenBank Number/References
Apoptosis autophagy-related genes	*bcl-2*	F: AGGACACGCAGTTCTCTTGG	QBA85626.1
		R: AACAAGACCCAGGATGCCAG	
	*jnk*	F: TGGTGCGTAACCGACCTAAC	KC900087
		R: ACTGGTCCAATGACTGGCTG	
	lc3c	F: CACGTTGCCTATCCTCGACA	XM_050842539.1
		R: GTCATCGTCCCTACACTCGC	
	caspase 8	F: TGGAGCGTCATGGTTCAGAC	AKS36884.1
		R: CAGACAAGCCACCACTGCTA	
	beclin1	F: GCCCATATACTGTGGCGAGG	MH173046.1
		R: CCAGGTCAAAGAGCCCAGTT	
Oxidative stress-related genes	mas	F: GACGATGGTTATGGAGTGTCCT	[26]
		R: GAGGAGCTCTTTTTGCTGGAC	
	*gpx*	F: GGCTGGACACCCTAGACAAC	FJ617305.1
		R: TAAGGGCCGTCACAAGGAAC	
	keap1	F: AGGCATCTTCATTGTGGGGG	XP_027210665.1
		R: GTTACCAACGACCACCGAGT	
	p62	F: ACAGACGCCAAGTACCAAGG	XM_050829992.1
		R: AGGCTCACTCGTCTCCTGAT	
	*il-16*	F: AGAGGTTGTTCTTGTGCTGTCC	MG182159.1
		R: ACGAGGGTAATGGTGAATGGAG	
Immune-related genes	*litaf*	F:TAAAGGCAAGGGAGGCTTCG	KF892539.1
		R:GAATGGAGCTTGAGGTGGCA	
	hsp90	F: TCACCAACGACTGGGAGGAT	XM_050873094.1
		R: CAGGAAGAGGAGTGCCCTGA	
	propo	F: CCATCCCTTCCTGCTTACCA	EF493829.1
		R: CTCCATCACAAACCCTAACGACTT	
Internal reference	*β-actin*	F: AGCGCAAGTACTCCGTCTGGAT	SRR9599542
		R: AATGGCAGGGCCAGACTCAT	

## Data Availability

The data that support the findings of this study are available from the corresponding author upon reasonable request.
